# Is increased “stay away from bed” time associated with improved clinical rehabilitation outcomes in Japanese rehabilitation hospitals? A prospective observational study and clinical practice

**DOI:** 10.1007/s40520-019-01269-5

**Published:** 2019-07-20

**Authors:** Ichiro Murayama, Tsuyoshi Asai, Shogo Misu, Masaya Yamauchi, Azumi Miura, Takeshi Ikemura, Takahiro Takehisa, Yozo Takehisa

**Affiliations:** 1 Department of Occupational Therapy, Heisei Rehabilitation College, 2-26, Tsutonishiguchi, Nishinomiya, Hyogo 663-8231 Japan; 2 Department of Physical Therapy, Faculty of Rehabilitation, Kobe Gakuin University, 518 Ikawadanicho, Arise, Nishi-ku, Kobe, Hyogo 651-2180 Japan; 3 Department of Physical Therapy, Faculty of Nursing and Rehabilitation, Konan Women’s University, 6-2-13, Morikita-machi, Higashinada-ku, Kobe, Hyogo 658-0001 Japan; 4 Department of Community Health Sciences, Kobe University Graduate School of Health Sciences, 7-10-2, Tomogaoka, Suma-ku, Kobe, Hyogo 654-0142 Japan; 5 Higashiura Heisei Hospital, 1867, Kuruma, Awaji, Hyogo 656-2311 Japan; 6 Tamagawa Hospital, 5-31-1, Kokuryo, Cyohu, Tokyo 182-0022 Japan; 7 Hakuai Memorial Hospital, 9, Soden, Katsura, Tokushima-shi, Tokushima, 770-8023 Japan; 8 Setagaya Memorial Hospital, 2-30-10, Noge, Setagaya-ku, Tokyo, 158-0092 Japan

**Keywords:** Activities of daily living, Bed rest, Hospitals, Length of stay, Rehabilitation

## Abstract

**Background:**

A comprehensive team approach for increasing stay away from bed time (SaB-time) called CASaB was conducted at multiple rehabilitation hospitals.

**Aims:**

The aim of the present study was to investigate the association between SaB-time and clinical rehabilitation outcomes (CROs) before introducing CASaB (observational phase), and comparing CROs before and after CASaB (CASaB phase).

**Methods:**

This prospective observational study included patients who were admitted to nine rehabilitation hospitals, with complete data. The final analysis included 197/229 patients in the observation phase, and 229/256 patients in the CASaB phase. We first tested whether SaB-time was positively associated with CROs in an observational study, then compared CROs before and after CASaB.

**Results:**

In the observation phase, longer SaB-time was significantly associated with greater rehabilitation efficiency (REy) after adjusting for confounders (standardized *β* = 0.20, *p* = 0.007). In a comparison of CROs before and after CASaB, the length of hospital stay during the CASaB phase was significantly shorter than during the observational phase (61.5, 57.6–65.4 days vs 75.6, 71.4–79.9 days, *p* < 0.001), and the REy after CASaB was significantly greater than that before the CASaB (0.38, 0.33–0.42/day vs 0.28, 0.25–0.33/day, *p* = 0.006).

**Discussion:**

The current results suggest that increasing SaB-time may help the recovery of functional abilities, particularly for patients in rehabilitation hospitals.

**Conclusions:**

The CASaB provides a method for improving the recovery efficiency of patients in rehabilitation hospitals.

## Introduction

According to data reported in a white paper on Japan’s aging society in 2017, the number of adults aged over 65 years was estimated at 35.2 million, comprising 27.3% of the population [1]. Accordingly, the number of older adults requiring care and support from others has increased in line with population changes in recent years, and the economic burden on the long-term-care system has consistently increased [1]. Given this situation, the role of rehabilitation hospitals in helping patients efficiently recover activities of daily living (ADL) has grown substantially, and various types of new clinical practice are required.

An important objective of rehabilitation hospitals is to prevent deconditioning as a result of inactivity. To accomplish this objective, it is important for rehabilitation hospitals to maintain the patient’s physical activity at a certain level during their stay [2-5]. However, in many cases, a range of factors (e.g., limited mobility, cognitive impairment, improper time management by patients, restrictions on going outside, and delirium) may cause the duration of time spent in bed to increase, further reducing the patient’s physical activity levels [6-9].

Many therapists in rehabilitation hospitals consider improving “stay away from bed (SaB)” time as essential for recovery of patients’ physical functions and preventing physical deconditioning. Increased SaB-time may lead to increased physical activity, which can aid efficient recovery of clinical rehabilitation outcomes (CROs) [10-12]. In the current study, we propose a new clinical practice for increasing SaB-time and improving CROs. To our knowledge, no previous study has investigated whether increased SaB-time is associated with positive changes in CROs. Thus, we first tested whether SaB-time was positively associated with CROs in an observational study. We then introduced a comprehensive approach for SaB-time (CASaB) in nine rehabilitation hospitals, and compared CROs before and after CASaB.

## Methods

### Setting and study subjects

**Fig. 1:**
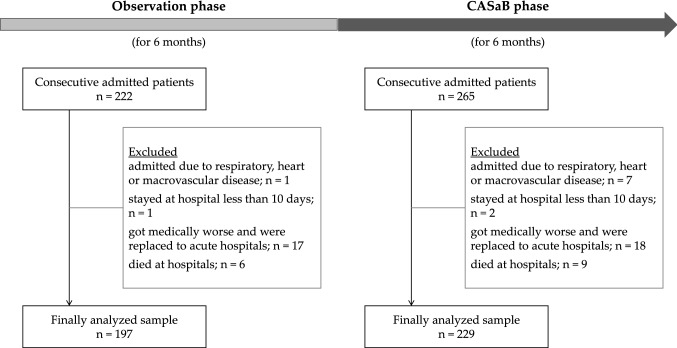
Diagram of study flow

This study used a multicenter design (at nine rehabilitation hospitals) consisting of two phases (Fig. 1): an observational phase (first 6 months, April 2016 and September 2016) and a CASaB phase (next 6 months, October 2016 and March 2017; see the “Comprehensive team approach for SaB-time” subsection for more details). The clinical observational data were collected in both phases. We included patients with complete data, who were admitted to the rehabilitation hospitals. We excluded from the analyses (1) patients who were instructed by a doctor to remain sedentary in bed, (2) patients who stayed in hospital for less than 10 days (e.g., those in respite care), (3) patients whose medical conditions worsened, and who were transferred to an acute hospital, and (4) patients who died in hospital. In the observational phase, 222 patients were admitted to hospital, and the final analyzed sample consisted of 197 patients. In the CASaB phase, 265 patients were admitted to the hospital, and the final analyzed sample consisted of 229 patients.

Demographic and clinical data such as age, sex, and discharge destination were collected from medical records. In the Japanese health insurance system, rehabilitation patients are categorized into five groups according to the disease causing hospitalization: (1) cerebrovascular disease and similar conditions; (2) locomotor disorder (post-operative for conditions including femoral neck fracture, total knee arthroplasty, compression fracture, degenerative lumber spondylosis); (3) disuse syndrome; (4) heart or macrovascular disease; and (5) respiratory disease. Patients categorized in the disuse syndrome group were patients who showed lower ADL ability due to the increased rest time in bed for treatment after the occurrence of a medical disease or surgical operation. Patients with heart or macrovascular disease, and respiratory disease were not included in this study, because there was only a small number of patients with these diseases (*n* = 4). Thus, our subjects were grouped into the remaining three groups. We measured subjects’ height, weight, mini-mental state examination (MMSE) scores [13], and grip strength. The study was carried out in accord with the principles of the Declaration of Helsinki. The Research Ethics Committee of Heisei hospital approved the study. Informed consent was obtained from all participants prior to participation.

### Assessment of SaB-time

In the present study, we defined SaB as “remaining in any postural condition other than lying down (e.g., sitting and standing)”. We included (1) the time spent in rehabilitation and (2) the time spent engaging in daily activities, such as eating, bathing and toileting, into SaB-time. The SaB-time of each patient was measured at intervals of approximately 30 min or less. An original SaB management sheet produced by each hospital was used to compute the SaB-time at the following time points:

1. The time at which patients received rehabilitation (physical, occupational, and speech therapies).
2. The time at which nurses and care workers completed regular rounds of patients on the hospital floors.
3. The time at which a SaB special facilitator (see the “Comprehensive team approach for SaB-time” for details) completed regular rounds of patients on the hospital floors.

### Assessment of clinical rehabilitation outcomes

The FIM is an assessment tool designed to evaluate the functional status of patients throughout the rehabilitation process [14]. FIM scores reflect the degree of disability, varying according to the patients’ ability to perform ADL. FIM scores include 18 categories, focusing on motor and cognitive function, and ranging from 0 to 126. Each category or item is rated on a seven-point scale (1 =  < 25% independence; total assistance required, 7 = 100% independence). FIM scores were measured at admission and discharge, and the following FIM score-based values and length of hospital stay (LOS) were used as CROs in the present study.

Absolute functional gain (AFG) was defined as the absolute difference between FIM scores at admission and discharge [14].

Rehabilitation efficiency (REy) was defined as the average increase in the FIM scores per day, and was calculated as AFG divided by LOS [14].

### Comprehensive team approach for SaB-time (CASaB, Fig. 2)

**Fig. 2:**
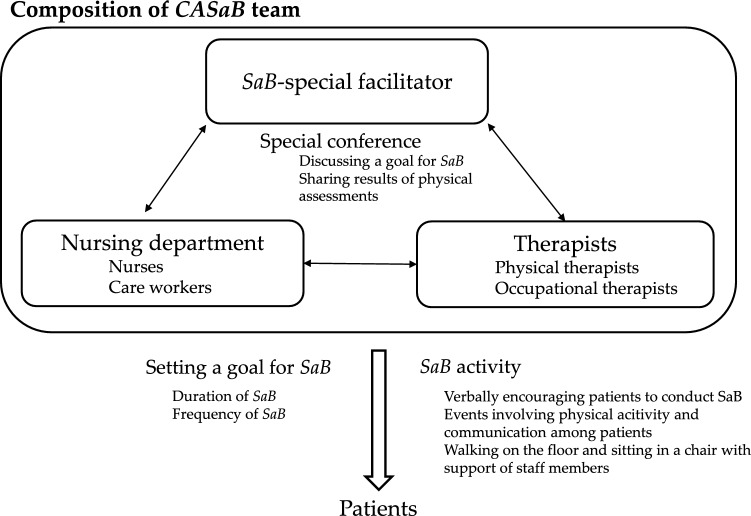
Comprehensive team approach for increasing “stay away from bed” time. *CASaB* comprehensive team approach for increasing “stay away from bed” time. *SaB* stay away from bed

An outline of the CASaB is shown in Fig. 2. The CASaB included the placement of a SaB special facilitator in the rehabilitation ward in hospital. The SaB special facilitator sets a SaB goal based on each patient’s condition, discussing the matter with the nurse and the therapist who were in charge of the patient. The SaB special facilitator provided information regarding the SaB goals (duration and frequency of SaB) to the nursing department (nurse and care workers). In addition, the CASaB team provided SaB activities to patients, such as (1) verbally encouraging patients regularly to perform SaB or (2) planning an event that involved physical exercise and interaction among patients. The condition of each patient was assessed in a special conference involving the SaB special facilitator, therapists, nurse and care workers, and, if necessary, the goal was reset. The CASaB can be easily conducted in a range of facilities because the tasks involve a relatively low burden for staff members. The CASaB was adopted in nine hospitals. Each patient typically underwent 1 or 2 h of physical and occupational therapy per day during their hospital stay, depending on their physical condition.

### Statistical analysis

All statistical analyses were carried out using commercially available software (JMP13.2.0; SAS Institute Japan, Tokyo, Japan). The background characteristics of patients between the observational phase and the CASaB phase were compared using unpaired *t* tests or *χ*^2^ tests. To explore the relationship between the change in CROs and the SaB-time, single linear regression analyses were performed, including the FIM score at admission, FIM score at discharge, AFG, LOS, and REy as dependent variables, and SaB-time as an independent variable. These analyses were performed among patients in the observation phase. In addition, to confirm the association after adjustment for potential confounding factors, multiple regression analyses were performed. Age, sex, disease causing hospitalization, MMSE, hand-grip strength, and FIM score at admission were included in the model as covariates.

Next, we ascertained whether SaB-time was longer in the CASaB phase than the observational phase using an unpaired *t* test. CROs (FIM score at admission, FIM, AFG, LOS, and REy) were then compared between the CASaB phase and the observational phase using unpaired *t* tests. Analyses of covariance (ANCOVA) adjusted for age, sex, the disease causing hospitalization, MMSE, hand-grip strength, and FIM score at admission were performed to compare CROs, except FIM score at admission between the CASaB phase and the observational phase. Each of the CROs except for FIM score at admission was included as a dependent variable, and the phase (CASaB phase or observational phase) was included as an independent variable. Finally, sub-group analyses were conducted according to the disease causing hospitalization (cerebrovascular disease and similar conditions, locomotor disorder, and disuse syndrome). In these analyses, the comparisons described above (unpaired *t* tests and ANCOVA) were performed in each sub-group. The level of significance for all analyses was set at *p* < 0.05.

## Results

### Characteristics

The patients’ characteristics are shown in e of patients in the observational phase (mean ± standard deviation [SD]) was 82.0 ± 9.6 years, and the range was 53–103 years. The age of patients in the CASaB phase was 79.0 ± 9.6 years (range 50–99 years). The results of the comparison using an unpaired *t* test revealed that the average age of patients in the observational phase was greater than that in the CASaB phase (*p* = 0.001). The MMSE score of patients in the observational phase (21.6 ± 5.9) was significantly lower than that in the CASaB phase (23.0 ± 6.3, *p* = 0.026). The hand-grip strength of patients in the observational phase was 16.2 ± 7.1 kg, and that in the CASaB phase was 23.0 ± 6.3 kg (*p* = 0.056). No significant differences were observed in the sex ratio, disease causing hospitalization, or discharge destination.

### The observational association between CROs and SaB-time

**Fig. 3:**
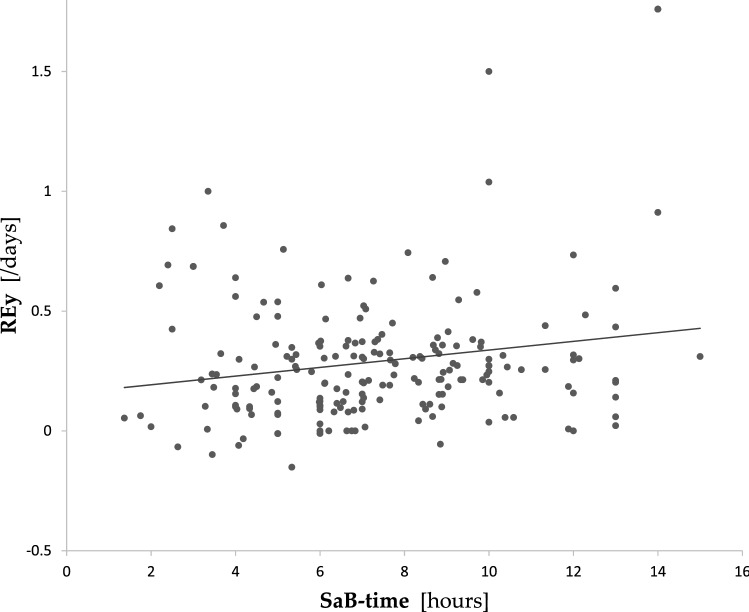
Results of a single linear regression analysis between stay away from bed time and rehabilitation efficiency in patients in the observation phase. Rehabilitation efficiency was calculated as the AFG (FIM score at discharge − FIM score at admission) divided by LOS. REy was significantly correlated with SaB-time (*y* = 0.156 + 0.018*x*, *r* = 0.188, *p* = 0.008). *SaB-time* stay away from bed time, *AFG* absolute functional gain, *FIM* functional independence measure, *LOS* length of hospital stay, *REy* rehabilitation efficiency

Longer SaB-time was significantly associated with higher FIM score at admission (*r* = 0.30, *p* < 0.001) and FIM score at discharge (*r* = 0.33, *p* < 0.001). However, these significant associations were diminished in the multiple regression analyses. There was no significant association between SaB-time and AFG. Longer SaB-time was significantly correlated with the higher REy (*y* = 0.156 + 0.018*x*, *r* = 0.188, *p* = 0.008, Fig. 3). This relationship was observed after adjusting for age, sex, disease causing hospitalization, MMSE, hand-grip strength, and FIM score at admission in the multiple regression analysis (standardized *β* = 0.20, *p* = 0.007, adjusted *R*^2^ for the whole model = 0.151).

### Comparisons between the observational phase and the CASaB phase

The results of the unpaired *t* test revealed that SaB-time in the CASaB phase (mean 8.2, 95% CI 7.7–8.7 h) was significantly longer than that in the observational phase (7.3, 6.9–7.7 h, *p* = 0.008, Table 1). Table 2 represents the results of comparisons of CROs between the observational phase and the CASaB phase. The FIM score at admission during the CASaB phase (79.7, 76.4–82.9) was greater than that during the observational phase (74.1, 70.7–77.5). FIM scores at discharge in patients during the CASaB phase (100.5, 97.5–103.4) were also greater those that during the observational phase (93.3, 89.6–96.9). However, no significant differences in FIM score at discharge were observed in the ANCOVA. LOS during the CASaB phase was significantly shorter (61.5, 57.6–65.4 days) than that during the observational phase (75.6, 71.4–79.9 days, *p* < 0.001). REy in patients during the CASaB phase (0.38, 0.33–0.42/day) was significantly greater than that during the observational phase (0.28, 0.25–0.33/days, *p* = 0.006). Significant differences in LOS and REy were observed after adjustment for age, sex, disease causing hospitalization, MMSE, hand-grip strength, and FIM score at admission (*p* < 0.001 and *p* = 0.016, respectively).

**Table 1:** Characteristics of patients in the observation phase and the CASaB phase

Variables	Observation phase (*n* = 197)	CASaB phase (*n* = 229)	*p* value
Age (years)	82.0 ± 9.6 [53–103]	79.0 ± 9.6 [50–99]	0.001
Females, *n* (%)	118 (59.9)	141 (61.6)	0.766
Height (m)	1.53 ± 0.10	1.53 ± 0.10	0.739
Weight (kg)	47.6 ± 10.8	49.5 ± 11.9	0.076
BMI (kg/m^2^)	20.3 ± 3.7	21.0 ± 4.0	0.053
MMSE score	21.6 ± 5.9	23.0 ± 6.3	0.026
Hand-grip strength (kg)	16.2 ± 7.1	23.0 ± 6.3	0.056
Medical condition group, *n* (%)			0.323
Cerebrovascular disease and similar conditions	36 (18.3)	35 (15.3)	
Locomotor disorder	61 (31.0)	64 (28.0)	
Disuse syndrome	100 (50.8)	130 (56.8)	
SaB-time (h)	7.3 ± 2.8	8.2 ± 3.7	0.008
Discharge destination, *n* (%)			0.323
Home	140 (71.1)	173 (75.6)	
Facility/hospital	57 (28.9)	56 (24.4)	

Values are means ± standard deviation or percentages. *P *values were calculated using unpaired *t* tests or *χ*^2^ tests between two samples

*MMSE* mini-mental state examination, *SaB-time* stay away from bed time

**Table 2:** Comparisons of rehabilitation outcomes between the observational phase and the CASaB phase in all patients and subgroup analysis by medical condition

Variables	Observational phase		CASaB phase		*p* value (simple comparison)	*p* value (after adjustment)
	Mean	95% CI	Mean	95% CI		
All patients						
FIM score at admission	74.1	70.7–77.5	79.7	76.4–82.9	**0.020**	–
FIM score at discharge	93.3	89.6–96.9	100.5	97.5–103.4	**0.002**	0.107
AFG	19.2	17.2–21.2	20.8	18.7–22.9	0.280	0.107
LOS (days)	75.6	71.4–79.9	61.5	57.6–65.4	** < 0.001**	**< 0.001**
REy (/day)	0.28	0.25–0.33	0.38	0.33–0.42	**0.006**	**0.016**
Cerebrovascular disease and similar conditions						
FIM score at admission	79.8	71.9–87.6	77.5	69.6–85.4	0.682	–
FIM score at discharge	103.1	96.1–110.1	101.1	94.6–107.6	0.672	0.997
AFG	23.3	18.4–28.3	23.6	18.9–28.3	0.937	0.997
LOS (days)	86.6	72.3–100.9	77.1	62.4–91.9	0.351	0.233
REy (/day)	0.31	0.24–0.38	0.38	0.30–0.46	0.201	0.185
Locomotor disorder group						
FIM score at admission	74.1	71.4–80.8	85.5	81.0–89.9	**0.005**	–
FIM score at discharge	97.1	92.2–102.0	105.3	101.7–109.0	**0.007**	0.684
AFG	20.9	18.1–23.7	19.9	17.2–22.5	0.579	0.684
LOS (days)	70.9	66.2–75.6	54.2	49.7–58.6	**< 0.001**	**< 0.001**
REy (/day)	0.34	0.27–0.40	0.39	0.33–0.46	0.248	0.209
Disuse syndrome group						
FIM score at admission	67.4	61.2–73.6	69.1	63.8–74.5	0.674	–
FIM score at discharge	81.3	74.5–88.1	90.3	84.2–96.3	**0.049**	**0.035**
AFG	13.8	10.4–17.3	21.1	16.4–25.8	**0.014**	**0.035**
LOS (days)	76.9	69.2–84.6	67.8	61.7–73.9	0.063	0.056
REy (/day)	0.20	0.15–0.24	0.33	0.25–0.42	**0.006**	**0.016**

*P *values for simple comparisons were calculated using unpaired *t* tests, and *p *values after adjustments were from the results of analyses of covariance adjusted for age, sex, MMSE, hand-grip strength, and FIM score at admission. Analyses of covariance for all patients also included medical condition group as a covariate

*CASaB* comprehensive team approach for SaB-time, *SaB-time* stay away from bed time, *CI* confidence interval, *FIM* functional independence measure, *AFG* absolute functional gain, *LOS* length of hospital stay, *REy* rehabilitation efficiency

### Comparison of disease causing hospitalization subgroups

We conducted unpaired *t* tests and ANCOVA in each disease causing hospitalization subgroup (cerebrovascular disease and similar conditions, locomotor disorder, and disuse syndrome; Table 2). In the cerebrovascular disease and similar conditions, no significant differences were observed in any CROs. In contrast, in the locomotor disorder group, LOS was significantly shortened during the CASaB phase (mean 54.2, 95% CI 49.7–58.6 days) compared with the observational phase (70.9, 66.2–75.6 days, *p* < 0.001) after adjusting for confounders. The FIM score at admission in this group during the CASaB phase (85.5, 81.2–89.7) was greater than that during observational phase (74.1, 71.3–81.0, *p* = 0.005). There were no significant differences in any other CROs. In the disuse syndrome group, FIM score at discharge, AFG and REy were significantly greater during the CASaB phase (FIM score at discharge: 90.3, 84.2–96.3; AFG: 21.1, 16.4–25.8; REy: 0.33, 0.25–0.42/day) compared with those during the observational phase (FIM score at discharge: 81.3, 74.5–88.1, *p* = 0.035; AFG: 13.8, 10.4–17.3, *p* = 0.035; REy: 0.20, 0.15–0.24/day, *p* = 0.016).

## Discussion

We investigated the association between SaB-time and CROs, and changes in CROs resulting from implementing the CASaB for patients in several rehabilitation hospitals. The results revealed that longer SaB-time was positively associated with greater improvements in CROs. SaB-time became longer as a result of implementing the CASaB, and positive changes in CROs were obtained. In addition, this positive change depended on the disease causing hospitalization (patients with disuse syndrome and locomotor disorder). To our knowledge, no previous studies have conducted this type of clinical practice in multiple facilities, representing a strength of the present study.

The current results revealed that longer SaB-time was associated with greater improvement of CROs, particularly rehabilitation efficiency. Importantly, several previous studies suggested that too much bed rest should be avoided in the early phase of recovery [11, 15]. Our results are in accord with these previous reports, suggesting that increasing SaB-time may help the recovery of functional abilities, particularly for patients in rehabilitation hospitals. As a potential explanation for this association, the increase in SaB-time may have been caused by an increase in physical activity in hospital, which is needed for physical recovery [10, 15-17]. However, because we did not measure physical activity directly, the causal relationship could not be confirmed [9]. Thus, further study is needed to clarify this issue. In addition, the current results indicated that an organized team approach is an effective way to increase SaB-time and promote improvement in CROs. In medical practice, various types of team approach are commonly implemented [18-21]. The current results suggest that a comprehensive team approach, including (1) placement of a special facilitator on the floor, (2) setting a goal for increased SaB-time, and (3) planning events to increase SaB-time, can provide an effective way of increasing SaB-time and promoting improvement in CROs. The present study involved a multicenter design including nine hospitals, and a large sample size. Therefore, the generalizability of our sample was partially confirmed, and the association between SaB-time and the CROs appeared to be robust.

The sub-group analyses revealed significant improvements of FIM scores by implementing CASaB in the disuse syndrome group. Patients with disuse syndrome exhibited hospital-related deconditioning and ADL limitations, which commonly occur during acute hospitalization due to injury, illness or both, but were not related to orthopedic or neurological diseases [22, 23]. Thus, most of the functional limitations among these patients may have been primarily caused by prolonged bed-rest time, and could potentially be improved by the CASaB. In the locomotor disorder group, LOS was shortened during the CASaB phase compared with the observational phase. Clinically, patients with locomotor disorder, such as hip fracture or compression fracture, tend to experience pain over a long period. Prolonged pain may make these patients inactive, delaying improvements of ADL function [24, 25]. Introducing the CASaB decreased the amount of unnecessary inactive time, which could have led to a shortened LOS. In contrast, there were no significant improvements of CROs in the cerebrovascular disease and similar condition group. Previous studies reported that increasing physical activity or the amount of rehabilitation led to greater improvements of CROs in stroke patients, which differed from the current results [12, 26]. The CASaB is designed to increase SaB-time, but this can include sitting in chairs or wheelchairs. To improve ADL functions in these patients, task-oriented approaches (e.g., increasing standing time or walking time) may be necessary because of the complex causes of ADL limitation (e.g., paralysis, sensory impairment, ataxia, equilibrium dysfunction, contracture, and cognitive impairment).

The current study involved several limitations that should be considered. First, the timing of SaB-time measurement was intermittent, and the intervals for some measurements were slightly larger. Thus, the measured SaB-time may have differed from the actual SaB-time. Second, the results may have been affected by biases, such as information bias, because CASaB members conducted the CASaB and also performed the assessment of SaB-time and CROs, and systematic bias, because the observation duration differed between the observation phase and the CASaB phase. Third, potential confounders may be related to limitations of the study design, particularly in the CASaB phase. We measured and compared CROs before and after the implementation of the CASaB as a clinical practice. Because some baseline characteristics of patients differed between two phases, we conducted multivariate analyses to adjust for these characteristics. However, it is possible that other potential confounders (e.g., concomitant medications) were also involved. Further studies using different designs should be conducted to confirm the effects of the CASaB on CROs, such as randomized controlled trials.
